# Hospital Barges for the Wounded

**Published:** 1915-10-16

**Authors:** 


### HOSPITAL BARGES FOR THE WOUNDED.

Long before the campaign began it was officially recognised that in certain circumstances inland transport by waterways might be made use of during war for the removal of the wounded, or for their transference from one centre to another. Various discussions and papers by officers of the Royal Army Medical Corps ventilated this subject, and a good many suggestions have been put forward as to the best ways of fitting up steamers, boats, or barges for hospital purposes of this sort. The canal system of England, upon which such an enormous capital expenditure had been lavished during the few years just before the advent of the railways, had been so neglected in consequence of the competition of rail transport, that such discussions almost seemed to some officers to have an air of unreality; it was difficult to believe that in England canal or river transport for the wounded would ever come within the range of practical politics. But in some Continental countries, and notably in the Low Countries, canal traffic is still an integral and even an important factor in transport economics. And our armies, as everyone knows, are operating in the southern marches of the Low Countries, just where the Flanders plain merges into the rolling country of Picardy and Artois in North-Western France. Even in Old France there are many navigable and navigated waterways in constant use; but Flanders is, of course, *par excellence* the country for water transport. Every battle almost that is reported in the districts held by the British is affected by one or other of the canals, as a glance at any map will indicate that it must be. Bethune, La Bassée, La Gorgue, Estaires, Merville, St. Omer, Calais, Dixmude, Ypres, Aire, and many other of the small towns and villages now household words amongst us, are all connected and served by canals or canalised rivers (Lawe, Yser, Lys, and others). In fact, it is possible to transport a soldier from the Front to the Base without his ever setting foot on dry land.

In these circumstances it is not astonishing that water transport should have been requisitioned for the removal of the wounded to the base hospitals. This method of water transport has several advantages over ambulance trains and motor-ambulances. It provides the absolute minimum of jolting and jarring to the patients; in fact the leisurely progress of a barge along an inland canal is about as smooth a motion as can possibly be achieved. The accommodation is far better, more complete, and more comfortable than that on a train, and much more so than that of a stretcher in a motor-ambulance on a *pavé* road. All such auxiliary aids to recovery, as cleanliness, good cooking, and fresh air, are far better attained on a hospital barge than on any other form of transport. The one disadvantage is the slowness of movement, which militates seriously against barge accommodation for any men with severe wounds such as are liable to require operative treatment. The wounded with whom the writer has conversed are loud in the praises of the comfort of their inland voyage, and in their appreciation of the excellent organisation and management of the flotillas of Red Cross barges. As a rule there is a tug which pulls a string of barges adapted to hospital requirements. One barge is reserved for the staff, and one for cooking and similar domestic functions; the remainder constitute the "wards."

The success of these floating hospitals seems to have been achieved by the local initiative of the Army Medical Service authorities; at least, no rumour of the co-operation of the Navy has reached home so far, though quite possibly the handymen may have advised or helped in the matter. Anyone interested in barge transport will find some suggestions for the conversion of barges into hospitals in one of the British Red Cross Society's manuals ("Hygiene and Sanitation," No. 4), from which the following is taken.

"The bilge water must first be pumped out and the bilge space cleaned and disinfected. The whole interior of the barge will require cleaning and repainting. Planks or wooden flooring must be laid in the well of the barge. . . . A roof frame, having an awning, is a desirable fixture as a protection against the weather. If possible, naval hospital cots, or wooden berths, should be fixed in the well; or . . . special fitments . . . . for holding stretchers may be used. . . . Box commodes containing pails will meet (lavatory) requirements. The pails should contain cresol . . . to be added to each pail twice daily. The box must be regularly scrubbed with soap and hot water, and kept screened or boarded off from the patients. Urine-pails are essential. They should stand on trays containing dry earth or cresol solution. . . . The contents of commodes and urine-pails should never be cast into the waterway, but buried in trenches on shore . . . It may be necessary to carry fresh water in quantity. Galvanised-iron storage tanks of sufficient size to hold twelve gallons per head per diem should be carried."

